# Artificial Intelligence–Enhanced Electrocardiography for the Diagnosis of Heart Failure With Preserved Ejection Fraction: A Systematic Review and Meta‐Analysis

**DOI:** 10.1155/crp/4994662

**Published:** 2026-06-04

**Authors:** Cian P. Murray, Hugo C. Temperley, Rob S. Doyle, Abdullahi Khair, Patrick Devitt, Solomon Asgedom

**Affiliations:** ^1^ Cardiology Department, Waterford University Hospital, Waterford, Leinster, Ireland, hse.ie; ^2^ Trinity Translational Medicine Institute, Trinity College Dublin, Dublin, Leinster, Ireland, tcd.ie; ^3^ Cardiology Department, Mater Misericordiae University Hospital, Dublin, Leinster, Ireland, mater.ie

## Abstract

**Background:**

Accounting for approximately 50% of heart failure, heart failure with preserved ejection fraction (HFpEF) is becoming increasingly common as populations age and multimorbidity grows. Diagnosis remains challenging, requiring multimodal testing with echocardiography, biomarkers and sometimes invasive haemodynamics. Artificial intelligence applied to the electrocardiogram (AI‐ECG) offers a low‐cost, scalable means of detecting HFpEF by extracting patterns beyond human interpretation.

**Methods:**

We conducted a systematic review and meta‐analysis in accordance with PRISMA, registered prospectively with PROSPERO. PubMed, Embase, Web of Science, and IEEE Xplore were searched to 1 August 2025 for studies evaluating AI/ML models applied to ECGs for the diagnosis of HFpEF or left ventricular diastolic dysfunction (LVDD). Eligible studies reported diagnostic performance compared with a recognized reference standard. Risk of bias was assessed with QUADAS‐AI. AUROC values were pooled using a logit transformation and random‐effects model, with results back‐transformed for interpretability.

**Results:**

Ten studies (2021–2025) met inclusion criteria, encompassing > 270,000 participants across diverse populations. Seven studies provided sufficient data for pooling, contributing 11 independent cohorts. The pooled AUROC was 0.84 (95% CI 0.78–0.88), indicating good discriminatory ability, though heterogeneity was extreme (I^2^ = 100%). Three additional studies reported diagnostic metrics without AUROC variance and were synthesized narratively. Risk of bias was moderate to high in several domains, driven by selective cohorts, inconsistent reference standards, and incomplete reporting.

**Conclusions:**

AI‐ECG shows promise for the detection of HFpEF, but the current evidence base is predominantly retrospective, methodologically heterogeneous, and limited by variable reference standards and insufficient external validation. No prospective, outcome‐based studies have yet established its clinical utility, and real‐world implementation remains untested.

## 1. Introduction

Heart failure with preserved ejection fraction (HFpEF) is now recognized as a major global health burden, accounting for up to half of all heart failure cases [1]. Its prevalence continues to rise in parallel with population ageing and increasing comorbidity rates, particularly obesity, hypertension, diabetes, and chronic kidney disease [2, 3]. HFpEF is associated with significant morbidity, including exercise intolerance, frequent hospitalizations, impaired quality of life, and increased mortality, with outcomes that approach those seen in heart failure with reduced ejection fraction (HFrEF) [1, 3].

Despite advances in diagnostic tools, HFpEF remains particularly difficult to diagnose in clinical practice. In contrast to heart failure with reduced ejection fraction (HFrEF), which can be identified relatively easily by reduced left ventricular systolic function, HFpEF requires a more nuanced, multimodal assessment [4, 5]. Current diagnostic pathways integrate symptoms, natriuretic peptide levels, echocardiographic indices of diastolic dysfunction and structural heart changes, and, when non‐invasive findings are inconclusive, invasive haemodynamic measurements such as pulmonary capillary wedge pressure or left ventricular end‐diastolic pressure. These pathways are resource‐intensive, variably available and prone to underdiagnosis or delayed recognition, particularly in community and primary care settings. The challenge is compounded by the nonspecific nature of HFpEF symptoms, which often overlap with common comorbid conditions, and by the frequent absence of congestion on examination at rest, resulting in substantial delays in timely diagnosis [1, 6].

For many years, HFpEF management was limited to symptom control and comorbidity treatment, with no proven disease‐modifying therapies. Recently, large trials have shown that sodium–glucose cotransporter 2 (SGLT2) inhibitors reduce hospitalization and improve quality of life in HFpEF (DELIVER, EMPEROR‐Preserved), and other therapies such as mineralocorticoid receptor antagonists and angiotensin receptor–neprilysin inhibitors show promise in selected groups [6–9]. These advances highlight the critical role of timely diagnosis in enabling early identification and initiation of effective therapies.

The 12‐lead electrocardiogram (ECG) is among the most widely available, inexpensive and routinely obtained investigations in cardiovascular medicine [10]. Yet, traditional visual interpretation has limited value in diagnosing HFpEF, as ECG changes are often subtle or nonspecific [11]. Advances in artificial intelligence (AI) and machine learning (ML) now allow extraction of latent patterns from ECG signals beyond human recognition [12]. In this review, AI is used as an umbrella term encompassing ML methods, including deep learning (DL), a subset of ML based on neural network architectures. AI‐ECG models have already demonstrated diagnostic and prognostic utility in HFrEF, with multiple meta‐analyses performed to date [13]. For example, Khan et al. reported a pooled sensitivity of 0.93 and a specificity of 0.95 across more than 218,000 patients, although their search ended in July 2023 and primarily included studies targeting systolic dysfunction [14]. Since then, several HFpEF‐focused studies have been published, yet no pooled evaluation has specifically addressed HFpEF. This represents an important gap, as detecting HFpEF from the ECG may be of even greater clinical value than HFrEF [15]. Whereas HFrEF can usually be confirmed readily with echocardiography, HFpEF diagnosis remains more complex, resource‐intensive and susceptible to delay.

Accordingly, we conducted a systematic review and meta‐analysis of AI‐ECG models for HFpEF detection. Our objectives were to assess the diagnostic potential of AI‐ECG for identifying HFpEF, to pool AUROC values where sufficient data were available and to guide future research by highlighting methodological limitations, sources of heterogeneity, and areas for improvement to support the eventual implementation of this technology in clinical practice.

## 2. Methods

### 2.1. Protocol and Registration

This systematic review was conducted in accordance with the Preferred Reporting Items for Systematic Reviews and Meta‐Analyses (PRISMA) guidelines. The protocol was prospectively registered with PROSPERO (registration number: CRD420251131560).

### 2.2. Eligibility Criteria

A PICO framework was used to define eligibility criteria. Eligible studies included the following:

1. Population: adults undergoing ECG testing, either in community, outpatient or hospital settings, with suspected or confirmed HFpEF or diastolic dysfunction.
2. Intervention/index test: application of AI or ML techniques to ECG signals (12‐lead, single‐lead or derived ECG formats).
3. Comparator/reference standard: diagnosis of HFpEF or left ventricular diastolic dysfunction (LVDD) as defined by the original study. Acceptable reference standards included guideline‐based definitions incorporating echocardiographic indices and/or invasive haemodynamic measurements, as well as pragmatic definitions based on preserved LVEF in the presence of clinical signs and symptoms of heart failure.
4. Outcome: diagnostic performance metrics (e.g. AUROC, sensitivity, specificity, accuracy, F1‐score) of AI‐ECG compared with the reference standard.

Studies were included if they reported original data on the diagnostic accuracy of AI‐ECG for HFpEF/LVDD. Exclusion criteria were studies limited to HFrEF or systolic dysfunction without HFpEF subgroup analysis, methodological papers without test set performance, review articles, editorials, case reports or abstracts.

### 2.3. Information Sources and Search Strategy

A comprehensive literature search was performed in PubMed (MEDLINE), Embase, IEEE Xplore and Web of Science from inception to 1 August 2025. Studies were restricted to those published in English due to resource constraints and the lack of validated translation for technical AI/ML reporting, which may limit interpretability and reproducibility. The full search strategy, including all keywords and Boolean operators, is provided in Supporting Information 1. To ensure completeness, we screened reference lists of all included studies and relevant reviews and manually searched grey literature.

### 2.4. Study Selection

All search results were imported into EndNote, and duplicates were removed. Two reviewers independently screened titles and abstracts, followed by full‐text review of potentially eligible studies against the predefined criteria. Disagreements were resolved by consensus or, if required, by adjudication from a third reviewer. The selection process is illustrated in a PRISMA flow diagram (Supporting Information 2).

### 2.5. Data Extraction

Two reviewers independently extracted study‐level data using a piloted form. Extracted variables included study design, country, setting, sample size, population characteristics, ECG acquisition type, AI/ML model architecture, training and validation approach, reference standard, and diagnostic performance metrics. Discrepancies were resolved by consensus or third‐party adjudication. Data management was performed using Covidence.

### 2.6. Quality Assessment

Methodological quality and risk of bias were assessed using the QUADAS‐AI tool, which extends the original QUADAS‐2 framework to the evaluation of AI studies in diagnostic test accuracy research. QUADAS‐AI evaluates risk of bias across four core domains—patient selection, index test, reference standard and flow/timing—while also incorporating AI‐specific considerations such as data handling, model training, validation strategy and risk of data leakage [16]. Applicability concerns were judged in parallel for patient selection, index test and reference standard. This approach provided a structured and AI‐specific framework for assessing methodological rigour and clinical relevance across the included studies.

### 2.7. Data Synthesis

Where sufficient data were available, we conducted a meta‐analysis of AUROC values. AUROC was selected as the primary summary metric because most included studies reported only AUROC, often without confusion matrices or sensitivity–specificity pairs at a defined threshold. This precluded the use of hierarchical bivariate models or summary ROC approaches, which require threshold‐level data. AUROC values were first logit‐transformed to stabilize variance and pooled using the Generic Inverse Variance method under a random‐effects model in RevMan 5.4. Standard errors were derived from reported confidence intervals, from case/control counts using the Hanley–McNeil method or from bootstrap estimates where applicable. Pooled results were then back‐transformed to the AUROC scale for interpretability, recognizing that confidence intervals become asymmetric after back‐transformation due to the bounded nature of AUROC values. We acknowledge that AUROC reflects discrimination only and does not capture calibration or clinical decision utility; however, it was selected as the primary metric due to inconsistent reporting of threshold‐level performance, calibration and alternative metrics such as precision–recall curves across studies.

Heterogeneity was assessed using the *χ* ^2^ test and quantified with the I^2^ statistic. Forest plots were generated to display results on the AUROC scale. For studies reporting multiple independent cohorts, internal and external validations were treated as separate datasets to avoid double counting. Where studies evaluated multiple model variants (e.g., 12‐lead vs. single‐lead ECG), the model judged to be most clinically representative or widely applicable was included in the main analysis, with alternative models summarized narratively.

Studies that did not report AUROC or did not provide sufficient information to derive variance estimates were excluded from quantitative synthesis but included in the systematic review. These were summarized narratively, with structured comparison by study design, reference standard and validation strategy to highlight methodological trends and identify areas for improvement in future research.

## 3. Results

### 3.1. Study Selection and Characteristics

**FIGURE 1:**
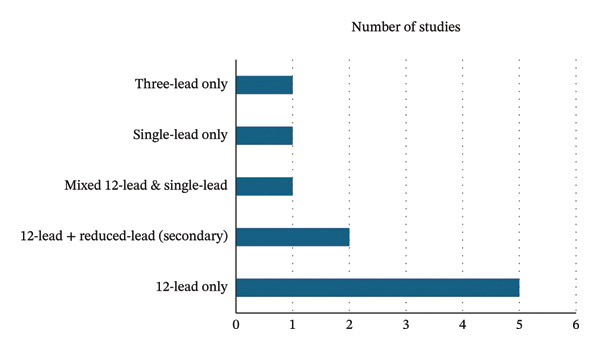
Bar chart demonstrating the distribution of ECG modalities used across included studies.

Our search identified 10 eligible studies evaluating AI‐ECG for the detection of HFpEF or LVDD [17–26]. Study‐level characteristics are summarized in Table 1. Published between 2021 and 2025 across Asia, Europe and North America, they included both highly selected cohorts (e.g. invasive haemodynamics, military conscripts, case–control series) and large consecutive hospital datasets. Sample sizes varied widely, from fewer than 200 patients to more than 270,000. Most studies evaluated standard 12‐lead ECGs exclusively (Figure 1) [17–19, 25, 26]. A smaller number used 12‐lead ECGs as the primary modality with additional reduced‐lead analyses [20, 23, 24]. One study evaluated single‐lead ECG alone and another focused solely on reduced‐lead configurations [21, 22]. Reference standards varied in evidentiary strength. Invasive haemodynamics (Gao et al. [18], Schlesinger at al. [26]) represented the most robust standards, while guideline‐concordant echocardiographic definitions were used in Kwon et al. [23], Lee et al. [24] and Unterhuber et al. [17]. Hong et al. applied the HFA‐PEFF score as a structured composite approach [19]. In contrast, Karabayir et al. used an ICD‐based HFpEF definition and Kavas et al. used a simplified preserved‐EF plus symptom‐based definition, both more prone to misclassification [20, 21]. Lin et al. and Kuznetsova et al. assessed LVDD rather than fully adjudicated HFpEF, representing related but non‐equivalent diagnostic targets [22, 25].

**TABLE 1:** Study characteristics of included studies.

First author (year)	Country	Setting	Population	Reference standard (HFpEF/LVDD)	Reference standard category/evidentiary strength	ECG type	Study design	Primary outcomes studied
Gao (2025)	China	Hospital/clinic	Adults (18–75) with LVEF > 50%, undergoing left ventricular catheterization; exclusion: AF, MI, ischaemia, pacemaker, etc.	HFpEF defined by LVEDP > 15 mmHg; or high‐risk for HFpEF defined as LVEDP > 12 mmHg + symptoms (dyspnoea, chest discomfort, fatigue, etc.)	Invasive haemodynamic/high	12‐lead	Prospective cohort validation of a CNN–LSTM deep learning model	Diagnostic performance of the CNN–LSTM deep learning model using ECGs to identify patients at risk for HFpEF
Hong (2025)	South Korea	Hospital/clinic	Consecutive patients who underwent TTE between January 2016 and December 2022. Patients excluded if they met any of the following criteria: LVEF < 50%, no NT‐proBNP, incomplete parameters for HFApPEFF score, lack of sinus rhythm	HFA‐PEFF score ≥ 5 defined as HFpEF	Composite diagnostic score/intermediate	12‐lead	Retrospective cohort study (training/validation/test split 7:1:2)	Primary: Discriminative ability of AI‐ECG model to detect HFpEF (AUROC). Secondary: Prognostic outcomes–cardiac death, HF hospitalization, composite outcome at 5 years
Karabayir (2024)	United States	Multicentre	Adults ≥ 18 years with ≥ 1 ECG; derivation cohort = 165,243 patients (1,078,198 ECGs), validation cohort = 42,880 patients (72,832 ECGs).	EF ≥ 50 and an ICD‐based HF diagnosis within 1‐year of an echo	Administrative coding with preserved EF/low	12‐lead and single lead (lead 1)	Retrospective derivation and external validation of AI model (multi‐class classification).	Discrimination of HF subtypes (rEF, mEF, HFpEF vs controls) by ECG‐AI models (AUC, sensitivity, specificity).
Kavas (2022)	Turkey	Hospital/clinic	61 volunteers multiple ECGs each (≥ 25 years; 60 HFrEF ECG, 60 HFpEF ECG, 60 healthy ECG; total 180 ECG recordings from repeat sampling)	HFpEF = LVEF ≥ 50% in a patient with clinical signs/symptoms of HF	Simplified preserved‐EF symptom‐based definition/low	3‐lead ECG (bipolar Lead I; right ankle, right & left wrist)	Prospective, single‐centre, diagnostic model development and validation study	Accuracy and classification performance (AUC, sensitivity, specificity, F1 score) of ML algorithms (k‐NN, SVM, Decision Trees, Ensemble) in diagnosing HFpEF, HFrEF, or healthy
Kuznetsova (2022)	Russia	Hospital/clinic	668 adults (≥ 18 yrs), consecutive outpatients or hospitalized patients at cardiology department; exclusions: severe LBBB, WPW, pacemaker rhythm, severe mitral stenosis, poor‐quality ECG/ECHO	Echocardiography with tissue Doppler per ASE/EACVI guidelines; LVDD defined as ≥ 3 of: (1) e′ septal < 7 cm/s or lateral < 10 cm/s, (2) LA volume index > 34 mL/m^2^, (3) E/e′ > 14, (4) TR velocity > 2.8 m/s	Guideline‐concordant echocardiographic LVDD surrogate/indirect‐high	Single‐lead smartphone‐case ECG (CardioQVARK device, lead I)	Prospective, cross‐sectional diagnostic accuracy study (development and validation cohorts)	Diagnostic accuracy of smartphone‐based ECG + ML for LVDD detection vs echocardiography (sensitivity, specificity, accuracy, repeatability)
Kwon (2021)	South Korea	Multicentre	Adults ≥ 15 years undergoing both ECG and echocardiography within 1 week, with preserved EF (≥ 50%). *N* = 34,103 patients (5756 HFpEF).	HFpEF defined as LVDD + preserved EF (≥ 50%) + HF symptoms/signs. LVDD criteria per ASE/EACVI: septal e’ < 7 cm/s or lateral e’ < 10; E/e’> 14; TR velocity > 2.8 m/s; LA volume index > 34 mL/m^2^. ≥ ½ criteria = abnormal.	Guideline‐concordant echocardiography with symptoms/high	12‐lead	Multicentre retrospective cohort study (two hospitals; internal + external validation)	Performance of deep learning model (DLM) to detect HFpEF from ECG vs reference echocardiographic diagnosis (AUC, sensitivity, specificity).
Lee (2024)	USA	Hospital/clinic	Adults ≥ 18 years with ECG and echocardiogram performed within 14 days (*n* = 274,710). No exclusion criteria.	Echocardiographic diastolic function and filling pressure (ASE/EACVI 2016 algorithm using e’, E/e’, LA volume index, TR velocity).	Guideline‐concordant echocardiographic LVDD/filling pressure surrogate/indirect‐high	12‐lead	Retrospective cohort study, large‐scale model development and validation.	Development and validation of an AI‐enabled ECG to classify diastolic dysfunction grades (≥ 1, ≥ 2, 3) and detect increased LV filling pressure vs echocardiography.
Lin et al. (2021)	Taiwan	Military health screening program	2206 young Asian military males, age 17–43 (mean 28)	Echocardiography (ASE/EACVI 2016 criteria): LVDD defined by any of: E/A ratio < 0.8, lateral e’ < 10 cm/s, or E/e’ > 14	Simplified echocardiographic LVDD surrogate/indirect‐intermediate	12‐lead	Cross‐sectional diagnostic study	Diagnostic performance of machine learning classifiers (RF, SVM, GBDT) using ECG features to detect left ventricular diastolic dysfunction (LVDD).
Schlesinger (2022)	United States	Hospital/clinic	4304 patients undergoing 6739 right heart catheterizations (Jan 2010–Oct 2020); adults 18–99 years;	RHC with mPCWP > 15 mmHg as the threshold for abnormal filling pressure	Invasive haemodynamic/high	12‐lead	Retrospective, model development and validation (with separate holdout cohort)	Discrimination ability of AI‐ECG to detect elevated mPCWP > 15 mmHg
Unterhuber (2021)	Germany	Hospital/clinic	Derivation: 1884 patients with preserved LVEF (≥ 50%) who presented with exertional dyspnoea or suspected CAD. Validation: 203 patients in a prospective HF screening program at risk for HF (risk factors, preserved EF)	ESC guideline‐based diagnosis: HF symptoms/signs, elevated natriuretic peptides, and structural/functional echo abnormalities; invasive LVEDP/PCWP (> 15–16 mmHg) used in a subset for confirmation.	Guideline‐concordant HFpEF definition/high	12‐lead	Retrospective derivation cohort with prospective external validation	Diagnostic performance of CNN to detect HFpEF vs controls (AUC, sensitivity, specificity, PPV, NPV)

*Note:* BNP = B‐type natriuretic peptide, TTE = transthoracic echocardiography, HFA‐PEFF = Heart Failure Association Pre‐test Assessment, Echocardiography and natriuretic peptide, Functional testing, and Final aetiology score, e′ = early diastolic mitral annular velocity, E/e′ = ratio of mitral inflow velocity to annular early diastolic velocity, AUROC/AUC = area under the receiver operating characteristic curve, ASE/EACVI = American Society of Echocardiography/European Association of Cardiovascular Imaging. NT‐proBNP, N‐terminal pro–B‐type natriuretic peptide.

Abbreviations: CAD, coronary artery disease; CNN, convolutional neural network; DLM, deep learning model; EF, ejection fraction; GBDT, gradient boosting decision tree; HFpEF, heart failure with preserved ejection fraction; HFrEF, heart failure with reduced ejection fraction; ICD, International Classification of Diseases; k‐NN, k‐nearest neighbours; LA, left atrium; LSTM, long short‐term memory network; LVDD, left ventricular diastolic dysfunction; LVEDP, left ventricular end‐diastolic pressure; LVEF, left ventricular ejection fraction; ML, machine learning; mPCWP, mean pulmonary capillary wedge pressure; NPV, negative predictive value; PCWP, pulmonary capillary wedge pressure; PPV, positive predictive value; RF, random forest; RHC, right heart catheterization; SVM, support vector machine; TR, tricuspid regurgitation.

### 3.2. AI/ML Model Characteristics

Index model details are summarized in Table 2. Inputs included raw ECG waveforms or engineered feature sets, with or without clinical variables. DL (e.g. CNNs, ResNet, CNN–LSTM) predominated in recent work, while earlier studies employed traditional classifiers such as k‐nearest neighbours or random forest. Training sizes ranged from 61 patients in small pilot studies to > 1 million ECGs in registry‐based models. Validation strategies varied: some used independent external cohorts (e.g., Kwon et al. [23], Karabayir et al. [20], Unterhuber et al. [17]), while others relied on internal splits or single‐centre testing.

**TABLE 2:** AI/ML model details.

First author (year)	Index test/model name	Inputs used	Preprocessing/Representation	Model architecture/Approach	Training sample size	External validation (yes/no)/calibration (yes/no)	Threshold/output type
Gao (2025)	CNN–LSTM deep learning model (DLM) for HFpEF risk	12‐lead ECG only (10‐s digital signals, 500 Hz; no clinical covariates)	Raw XML amplitudes ⟶ denoising, matrix transformation, grouped into 5000 × 6 precordial‐lead matrices	Hybrid convolutional neural network + long short‐term memory (CNN‐LSTM); ∼163k parameters; implemented in TensorFlow	Cohort A: 238 patients (invasive LVEDP reference)	Yes—prospective external validation in Cohort B: 117 patients (independent hospital cohort, 2023‐24) /No	Binary high‐ vs low‐risk classification; cut‐off LVEDP > 12 mmHg
Hong (2025)	AI‐enabled ECG model to predict HFpEF (HFA‐PEFF ≥ 5)	ECG‐only; 12‐lead, 10‐s digital ECGs, sinus rhythm; 500 Hz; Philips PageWriter TC70/TC50/TC30/Trim III	Raw 1‐D time‐series waveforms stored as XML; 12‐lead, 10 s at 500 Hz	1‐D DenseNet‐121 CNN (single‐centre development)	Total *N* = 13,081; Training *n* = 9156; Validation *n* = 1308; (Test *n* = 2617)	No—internal hold‐out (single‐centre) with 7:1:2 split (train:val:test); test set reserved for evaluation; no external validation /No	Probability output with binary classification at Youden‐index threshold
Karabayir (2024)	ECG‐AI multiclass classifier for HF subtypes (12‐lead ResNet; companion Lead I ResNet version)	ECG‐only models (primary): raw digital 12‐lead, 10‐s recordings; separate single‐lead Lead I model. Clinical variables were only used in secondary fusion models (LightGBM).	Raw 1‐D time‐series ECG; export/decoding/parsing with resampling (details in supplement). Lead I input shape reported as 2500 × 1 (implies 10 s at ∼250 Hz).	1‐D ResNet CNN (batch normalization, dropout, Adam optimizer); same approach for 12‐lead and Lead I variants.	Derivation (AHWFB): 1,078,198 ECGs from 165,243 adults; split 80/10/10 (train/val/holdout) at patient level with balanced outcomes.	Yes—internal hold‐out (‘holdout‐2’): 21,957 ECGs/3305 pts (unseen during development). External adult validation (UTHSC): 72,832 ECGs/42,880 pts (independent system). /No	Multiclass softmax probabilities (rEF, mEF, HFpEF, control). Class‐wise ‘one‐vs‐rest’ binary metrics reported; specific thresholding method not reported (NR).
Kavas (2022)	Medical decision support system for classifying HFrEF vs HFpEF vs healthy using ECG features	ECG‐only (no clinical variables)	Biopac MP36 acquisition; 10 s recordings at 200 Hz; filtering: Chebyshev Type II band‐pass 0.25–100 Hz (60 dB), 50 Hz notch (49–51 Hz, 60 dB), moving‐average; periodogram features, 37 engineered features (21 time‐domain + 16 AR from Yule‐Walker and Burg).	Traditional ML on engineered features; classifiers evaluated: k‐NN (Euclidean), SVM (cubic polynomial), Decision Tree, Bagged Trees	61 volunteers; 180 ECG recordings total; training set ≈ 144 recordings (80%)	No—internal split only: stratified 80/20 by class (HFrEF/HFpEF/healthy) to create a balanced test set; no external validation /No	Multiclass label output (HFrEF/HFpEF/healthy); no probability thresholding reported
Kuznetsova (2022)	Smartphone‐case single‐lead ECG with spectral analysis (CardioQVARK) for LVDD screening	ECG‐only, single‐lead (lead I) recorded from fingers; 3‐min recordings; no clinical variables	1000 Hz single‐lead ECG; device analogue bandwidth 0.67–320 Hz; signal filtering/marking; continuous wavelet transform spectral features; time/amplitude indices; median per‐beat feature vectors	ML‐derived rule combining four ECG parameters (QTc, QRSfi, Tpeak, Toffs); thresholds derived via logistic regression/ML; integrated algorithm in device (no specific classifier named)	Development cohort phase 1: 418 included (from 446 recruited)	No—prospective internal validation (phase 2) in separate cohort from same centre: 250 included (paired repeat recordings to assess repeatability); no multicentre external validation /No	Binary output: LVDD present if QTc > 425 ms, QRSfi > 674 ms, Tpeak > 590 ms, and Toffs > 695 ms all exceeded; otherwise LVDD absent; rule integrated into device
Kwon (2021)	Ensemble deep learning model (DLM) for HFpEF detection	ECG + demographics (age, sex, weight, height)	Raw 12‐lead ECG at 500 Hz, 10 s recorded; middle 8 s used (first/last 1 s excluded for artefact); represented as 12 × 4000 2‐D numeric array	Convolutional neural network blocks (conv + batch norm + max‐pool + dropout) feeding a 256‐node layer; concatenated with demographic inputs; fully connected layers; sigmoid output; implemented in TensorFlow	Development set: 32,671 ECGs from 20,169 patients (Hospital A)	Yes—internal validation: 1979 ECGs from 1979 patients (Hospital A); External validation: 11,955 ECGs from 11,955 patients (Hospital B) /No	Probabilistic output (0‐1); binary classification using cut‐off chosen by Youden’s J on development data
Lee (2024)	AI‐enabled ECG for diastolic function grading and increased filling pressure (ResNet‐18)	ECG‐only; 12‐lead, 10‐s digital ECGs; 500 Hz (ECGs at 250 Hz up‐sampled to 500 Hz); no clinical variables	12 × 5000 time‐series matrix per ECG; split into five non‐overlapping 2‐s windows; average the window outputs	1‐D CNN based on ResNet‐18; multi‐class outputs (normal, grade 1–3) aggregated to binary (normal vs increased filling pressure); Adam optimizer, lr = 0.001, up to 20 epochs; model selected by validation AUC	Training *n* = 98,736; Validation *n* = 21,963; Test *n* = 98,763	No—internal hold‐out split (45/10/45 for train/val/test); additional evaluation in indeterminate‐by‐echo cohort *n* = 55,248; no external validation /No	Probability outputs; thresholds chosen by Youden index (e.g., 0.26 for increased filling pressure; 0.443 for grade ≥ 1; 0.264 for grade ≥ 2; 0.058 for grade 3)
Lin et al. (2021)	Machine‐learning classifiers (random forest, SVM with RBF kernel, gradient boosting decision tree) to predict LV diastolic dysfunction	ECG features only (26 conventional 12‐lead features: heart rate; lead II P‐wave duration; PR/QRS/QT intervals; P/QRS/T axes; R‐wave voltages in I, II, III, aVR, aVL, aVF; R and S in V1–V6); or ECG + 6 biological features (age, height, weight, waist circumference, systolic/diastolic BP)	Conventional ECG acquired on Schiller MS‐2015 or Philips TC70 with built‐in artifact/AC/baseline‐wander filters; min–max feature normalization; class imbalance handled with SMOTE; features (not raw waveforms) used as inputs	Traditional ML: RF (CART/Gini; number of trees tuned), SVM (RBF kernel; soft‐margin L2; γ and C tuned), GBDT (up to 100 iterations; max depth tuned); hyperparameters selected by grid search maximizing AUC‐PR; implemented in scikit‐learn	Total *N* = 2206; split 3:1 into train + validation vs test. Train + validation ≈ 1654 (3‐fold CV with balanced LVDD per fold); test ≈ 552; LVDD prevalence 4.26% (94/2206)	No—internal only: stratified 3:1 train/test; within train + validation, 3‐fold cross‐validation; no external validation /No	Binary LVDD classification; operating cut‐offs in testing chosen to target specificity ∼70%–80% (≈75%); classifier score threshold applied
Schlesinger (2022)	RHCNet (Right Heart Catheterization Network): deep learning model to infer elevated mean PCWP (> 15 mmHg) from 12‐lead ECG	ECG‐only, standard 12‐lead clinical ECGs; most sampled at 500 Hz (250 Hz ECGs upsampled to 500 Hz); no clinical variables as model inputs	Raw time‐series waveforms; ECGs with nonphysical values removed; each ECG matched to first same‐day right‐heart catheterization; labels binarized at mPCWP > 15 mmHg	Convolutional neural network with two‐stage training: pretrain on large ECG registry to predict ECG intervals, then fine‐tune for multitask classification (mPCWP, mPAP, PVR, CO); includes an unreliability score to flag inconsistent predictions	Development set 5390 procedures (train 4304; validation 546; test 540); pretraining registry 242,216 ECGs (unique patients)	No—patient‐level splits; internal train/validation/test plus independent holdout set of 1349 encounters from the same centre; no multicentre external validation /No	Probabilistic output for elevated mPCWP; binary classification relative to the 15 mmHg cutoff; operating thresholds varied (e.g., to target sensitivity 0.80 for PPV/NPV analyses)
Unterhuber (2021)	Convolutional neural network to detect HFpEF from baseline 12‐lead ECG	ECG‐only; baseline digital 12‐lead ECGs; 10 s recordings; no clinical variables	ECG divided into 2‐s segments for each lead; arranged into a 4 × 12 image grid; colour inversion, edge sharpening, and Canny edge detection; blank/non‐informative segments removed	CNN with four convolutional layers; each layer followed by ReLU and max‐pooling; sigmoid output; trained in Keras/TensorFlow‐GPU; RMSProp optimization; arithmetic mean of segment probabilities used for patient‐level prediction	Derivation cohort *n* = 1884 patients; total 77,558 ECG segments; split 50% train (*n* = 942), 30% internal validation (*n* = 565), 20% test (*n* = 377)	Yes—external prospective screening cohort *n* = 203 volunteers (independent cohort) in addition to internal held‐out test set /No	Binary classification; probability averaged across segments; operating cut‐off set to 0.4 using Youden index in derivation and applied to validation

*Note:* ECG = electrocardiogram, SMOTE = synthetic minority oversampling technique, AUC‐PR = area under the precision–recall curve, AR = autoregressive, QTc = corrected QT interval, Tpeak = T‐wave peak time, Toffs = T‐wave offset time, RMSProp = root mean square propagation optimizer, XML = extensible markup language, val = validation, pts = patients, AUROC, area under the receiver operating characteristic curve.

Abbreviations: AHWFB, Atrium Health Wake Forest Baptist; AI, artificial intelligence; AUC, area under the curve; BP, blood pressure; CNN, convolutional neural network; CO, cardiac output; DLM, deep learning model; GBDT, gradient boosting decision tree; HFpEF, heart failure with preserved ejection fraction; HFrEF, heart failure with reduced ejection fraction; k‐NN, k‐nearest neighbours; LSTM, long short‐term memory; LVDD, left ventricular diastolic dysfunction; LVEDP, left ventricular end‐diastolic pressure; ML, machine learning; mPAP, mean pulmonary artery pressure; mPCWP, mean pulmonary capillary wedge pressure; NR, not reported; PCWP, pulmonary capillary wedge pressure; PVR, pulmonary vascular resistance; QRSfi, QRS fragmentation index; RBF, radial basis function; ReLU, rectified linear unit; ResNet, residual neural network; RF, random forest; RHCNet, right heart catheterization network; SVM, support vector machine; UTHSC, University of Tennessee Health Science Center.

### 3.3. Model Performance

Performance outcomes are summarized in Table 3. Reported AUROCs ranged from 0.73 to 0.92 across validation cohorts, with most studies falling in the 0.80–0.90 range. Sensitivities and specificities, when reported, typically balanced between 70% and 85%, though some models (e.g., Unterhuber et al. [17]) prioritized very high sensitivity at the cost of lower specificity. Several large studies (e.g., Lee 2024 [24], Hong 2025 [19]) also demonstrated prognostic utility, with positive AI‐ECG predictions associated with adverse outcomes such as increased risk of heart failure hospitalization and mortality. In contrast, smaller proof‐of‐concept studies (e.g., Kavas 2022 [21]) reported very high or near‐perfect performance, often without confidence intervals or external validation, raising concerns regarding overfitting and limited generalizability. Across studies, reporting of clinically relevant performance metrics was inconsistent. Few provided calibration measures, precision–recall analyses or externally validated decision thresholds. Notably, no included studies reported formal calibration assessment (e.g., calibration plots, slopes or Brier scores). While some studies reported predicted probabilities, PPV/NPV or selected operating thresholds (e.g., Youden index), these do not constitute calibration and do not assess agreement between predicted and observed risks.

**TABLE 3:** Model performance and outcomes.

First author (year)	Validation cohort type and size	AUROC (95% CI)	Sensitivity (%)	Specificity (%)	PPV	NPV	Other outcomes
Gao (2025)	External prospective validation (Cohort B), *n* = 117	Not reported	71.7	71.9	NR	NR	‐ Accuracy reported as 71.8%. ‐ DLM high‐risk group had higher diabetes prevalence (22.03% vs 11.86%, *p* < 0.01) and BMI (25.92 vs 24.22 kg/m^2, *p* < 0.01), and lower CCB use (11.76% vs 28.81%, *p* = 0.05).
Hong (2025)	Internal hold‐out test cohort (single centre), *n* = 2617	0.81 (0.79–0.82)	72	74	69	77	‐ Subgroups AUC 0.78–0.83 across age, BMI, HTN, DM; ‐ Sensitivity analyses: excluding intermediate HFA‐PEFF 2–4 ⟶ AUC 0.90 (0.88–0.91); ECG within 3 months ⟶ AUC 0.80 (0.79–0.82). ‐ Prognosis (test cohort): positive AI‐ECG associated with higher risk at 5 years—composite HR 6.33 (2.50–16.07), cardiac death HR 9.56 (1.24–73.53), HF hospitalization HR 5.91 (2.08–16.81).

Karabayir (2024)	Internal hold‐out (AHWFB adults), *n* = 3305 patients; 21,957 ECGs used for evaluation	12‐lead: 0.80 [0.78–0.82]; Lead I: 0.75 [0.73–0.77]	NR	NR	NR	NR	‐ DeLong (12‐lead vs Lead I) *p* < 0.01 (AHWFB).
	External validation (UTHSC adults), *n* = 42,880 patients; 72,832 ECGs	12‐lead: 0.73 [0.72–0.74]; Lead I: 0.74 [0.73–0.75]	NR	NR	NR	NR	‐ DeLong (12‐lead vs Lead I) *p* = 0.08 (UTHSC); AHWFB vs UTHSC comparison for 12‐lead *p* < 0.01.
Kavas (2022)	Internal split (single centre), 80/20 stratified by class; 61 volunteers, 180 ECG recordings total (60 HFrEF, 60 HFpEF, 60 healthy)	Per‐class AUC 0.938 for k‐NN with 37 features; AUC increased with 19 features; for 4‐feature model results described as ‘ideal’; no 95% CI reported	NR	NR	NR	NR	‐ Best reported overall accuracy 100% for k‐NN on internal split; multiclass task (HFrEF/HFpEF/healthy); no external validation.

Kuznetsova (2022)	Development cohort (Phase 1), *n* = 418—combined 4‐parameter rule (QTc > 425 ms, QRSfi > 674 ms, Tpeak > 590 ms, Toffs > 695 ms)	0.766	86	**70**	NR	NR	‐ Odds ratio 11.7 (2.7–50.9), *p* < 0.001; per‐parameter AUCs: QTc 0.648, QRSfi 0.698, Tpeak 0.676, Toffs 0.704.
	Prospective internal validation (Phase 2), *n* = 250; LVDD prevalence 12.4%	NR	95.6	**97.7**	NR	NR	‐ Diagnostic accuracy 96.5%; repeatability 98.8% (paired measures differed in 3 patients); algorithm not applicable in 3.5% due to poor ECG/ECHO quality.

Kwon (2021)	Internal validation (Hospital A), *n* = 1979	0.866 (0.850–0.883)	NR	NR	NR	NR	‐ Subgroup with initially non‐HFpEF but follow‐up echo: high‐risk vs low‐risk by DLM developed HFpEF 33.6% vs 8.4% over 24 months (log‐rank *p* < 0.001). ‐ Also reported AUC for asymptomatic LVDD detection 0.837 (0.805–0.870).
	External validation (Hospital B), *n* = 11,955	0.869 (0.860–0.877)	NR	NR	NR	NR	‐ Additional modality results (for context): six‐lead AUC 0.858 and single‐lead AUC 0.845 (external).

Lee (2024)	Internal hold‐out test set, *n* = 98,763 (increased filling pressure task)	0.911 (0.909–0.914)	83.2	82.9	58.1	94.5	‐ AUCs for diastolic grades: ≥ 1 = 0.847 (0.844–0.850, thr 0.443); ≥ 2 = 0.911 (0.909–0.914, thr 0.264); grade 3 = 0.943 (0.938–0.948, thr 0.058). ‐ Lead I model AUCs: 0.804 (≥ 1), 0.875 (≥ 2), 0.915 (grade 3). ‐ Mortality associations: HR 1.70 (1.645–1.757) in test cohort; HR 1.34 (1.298–1.383) in indeterminate‐by‐echo cohort
Lin et al. (2021)	Internal test set (single centre), 3:1 split of 2206 young Asian male adults; test *n* ˜ 552; LVDD prevalence 4.26%	RF (ECG‐only): 0.841; SVM (ECG + biological): 0.821; 95% CI NR	RF (ECG‐only): 81.0; SVM (ECG + biological): 85.7	≈75 (targeted operating point used to set cut‐offs)	NR	NR	‐ Accuracy for ML models > 70%; PR‐AUC used for tuning; SMOTE applied due to class imbalance; additional metrics (precision, F1) reported in‐text/tables

Schlesinger (2022)	Internal test set (development set), *n* = 540	0.80 (SD 0.02; 95% CI NR)	NR	NR	NR	NR	‐ Pretrained + fine‐tuned CNN (RHCNet) on raw ECG to infer mPCWP > 15 mmHg.
	Independent holdout set (same centre), *n* = 1349	0.79 (SD 0.01; 95% CI NR)	80 (operating point used for PPV/NPV analyses)	NR	HF cohort: 0.76 Transplant cohort: 0.19	HF cohort: 0.42 Transplant cohort: 0.92	‐ AUC reduced to 0.70 ± 0.06 in highest decile of model unreliability; ‐ Higher Brier error in unreliable subgroup; ‐ PPV/NPV shown as functions of sensitivity and prevalence. Code and weights released (RHCNet)

Unterhuber (2021)	Internal blinded test set (held‐out 20%), *n* = 377	0.92 (0.91–0.94)	98	63	NR	NR	‐ Cut‐off set by Youden index (0.4) to maximize sensitivity; patient‐level prediction averaged over segment probabilities
	External prospective screening cohort, *n* = 203	0.80 (0.74–0.86)	99	60	68	69	‐ High NPV supports screening/rule‐out use case; activation maps highlighted QRS/ST segments.

*Note:* AUROC = area under the receiver operating characteristic curve, HFA‐PEFF, Heart Failure Association Pre‐test assessment, Echocardiography and natriuretic peptide, Functional testing, and Final aetiology score, QTc, corrected QT interval, Tpeak, T‐wave peak time, Toffs, T‐wave offset time, SMOTE, synthetic minority oversampling technique, F1, F1 score. Bold values indicate the values of Specificity in %.

Abbreviations: AHWFB, Atrium Health Wake Forest Baptist; AUC, area under the curve; BMI, body mass index; CCB, calcium channel blocker; CI, confidence interval; CNN, convolutional neural network; DLM, deep learning model; HF, heart failure; HFpEF, heart failure with preserved ejection fraction; HFrEF, heart failure with reduced ejection fraction; HR, hazard ratio; LVDD, left ventricular diastolic dysfunction; mPAP, mean pulmonary artery pressure; mPCWP, mean pulmonary capillary wedge pressure; NPV, negative predictive value; NPV, negative predictive value; NR, not reported; OR, odds ratio; PPV, positive predictive value; PR, precision–recall; PR‐AUC, precision–recall area under the curve; QRSfi, QRS fragmentation index; RF, random forest; RHCNet, right heart catheterization network; SVM, support vector machine; UTHSC, University of Tennessee Health Science Center.

### 3.4. Quantitative Synthesis (Meta‐Analysis)

**FIGURE 2:**
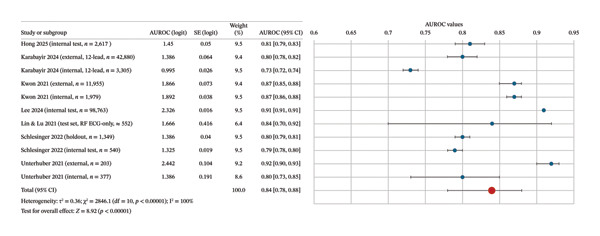
Forest plot of pooled AUROC values.

Seven studies reported AUROC values with sufficient detail to derive variance estimates, contributing 11 independent cohorts to the meta‐analysis. Pooled analysis under a random‐effects model yielded an AUROC of 0.84 (95% CI 0.78–0.88), indicating good overall discriminatory performance of AI‐ECG for HFpEF detection (Figure 2). Heterogeneity was extreme (I^2^ = 100%), reflecting major differences in populations, reference standards and modelling approaches. For example, studies using invasive haemodynamic definitions (e.g. Schlesinger et al. [26]) tended to report higher discrimination than those relying on pragmatic EF‐based or ICD‐coded diagnoses (e.g., Karabayir et al. [20]). Forest plots showed most point estimates clustered between 0.80 and 0.90, with some external validations reporting reduced performance (highlighting the challenge of reproducibility across settings.)

### 3.5. Narrative Synthesis

Three studies could not be included in the quantitative synthesis but provide important complementary insights. Gao et al. evaluated a CNN–LSTM model against invasive LVEDP, a strong physiological reference standard [18]. The model achieved balanced sensitivity and specificity of ∼72%–76%, but results were reported only as confusion matrix metrics, without AUROC or variance estimates. Kavas et al. [21] tested traditional classifiers on 3‐lead ECGs from 61 participants in a small case–control design. While crude accuracy was ∼80%, AUROC and confidence intervals were not reported, and HFpEF was defined only by EF ≥ 50% plus symptoms, raising concerns about reference standard validity. Kuznetsova et al. [22] explored smartphone single‐lead ECGs with a four‐parameter spectral rule, reporting AUROC values of 0.766 in the development set but not in validation and without confidence intervals.

### 3.6. Performance by ECG Modality

Most included studies evaluated standard 12‐lead ECGs, which provided the most robust evidence base and generally demonstrated good discrimination across validation cohorts. Evidence for reduced‐lead approaches was more limited and heterogeneous. In Karabayir et al., 12‐lead performance was modestly higher than single‐lead performance in the internal cohort (AUROC 0.80 vs 0.75), but similar in external validation (0.73 vs 0.74). Kwon et al. likewise reported slightly lower discrimination with reduced‐lead configurations compared with 12‐lead models. Lee et al. also demonstrated lower performance for a Lead I model than for the 12‐lead model across diastolic dysfunction thresholds (e.g., AUROC 0.804 vs 0.847 for grade ≥ 1 and 0.875 vs 0.911 for grade ≥ 2).

### 3.7. Risk of Bias

Risk of bias varied substantially across studies when assessed with QUADAS‐AI (Supporting Information 3a/3b). The most frequent concerns arose in patient selection, with several studies using case–control designs or highly selective cohorts (e.g., invasive catheterization populations, military males), while larger consecutive hospital‐based series were generally at lower risk. Reference standards ranged from guideline‐based echocardiography or invasive haemodynamics (low risk) to simplified EF thresholds or ICD‐coded diagnoses (high risk). Flow and timing were acceptable in most studies, though some allowed wide ECG–reference intervals or excluded a high proportion of patients. AI‐specific issues were common. Several studies did not report whether index test interpretation was blinded to the reference standard, and smaller datasets were particularly prone to overfitting, often without external validation. Risks of data leakage were present in studies using repeated random splits without clear separation of training and test data. Calibration, decision thresholds and confidence intervals were inconsistently reported. Applicability was lowest in small proof‐of‐concept studies using non‐standard devices or narrowly defined populations, and higher in large, consecutive hospital cohorts with standard 12‐lead ECGs and guideline‐based definitions. Several studies exhibited features strongly associated with overfitting and limited generalizability, including small sample sizes relative to model complexity, record‐level rather than patient‐level splitting, absence of external validation and lack of pre‐specified thresholds. Reports of near‐perfect or very high performance without external validation raise particular concern for overfitting or data leakage, reducing confidence in the reported diagnostic performance.

## 4. Discussion

This systematic review and meta‐analysis provides the first pooled evaluation of AI‐ECG for the detection of HFpEF. Across 10 eligible studies, AI‐ECG models demonstrated promising diagnostic performance, with a pooled AUROC of 0.84, indicating good discriminatory ability. However, this estimate should be interpreted with caution in light of the extreme heterogeneity observed (I^2^ = 100%), which limits the reliability and generalizability of the summary measure. This heterogeneity reflects substantial variation in study design, populations, reference standards and analytic approaches. In particular, differences in case definitions, model architectures and validation strategies suggest that the pooled AUROC represents an average across diverse clinical and methodological contexts, rather than a consistently reproducible level of performance. Additionally, this pooled AUROC does not constitute evidence of clinical utility, nor does it reflect performance within prospective or real‐world clinical pathways.

HFpEF is particularly challenging to diagnose in clinical practice. Unlike HFrEF, which can be identified relatively easily through reduced systolic function on echocardiography, HFpEF requires a complex, multimodal assessment incorporating symptoms, biomarkers, echocardiographic indices of diastolic function and sometimes invasive haemodynamics [1, 3]. These pathways are resource‐intensive, inconsistently available and often delayed in community settings [1, 6]. Symptoms are frequently nonspecific and overlap with common comorbidities, while congestion may be absent at rest, further obscuring diagnosis [1, 6]. In this context, a rapid, inexpensive and universally available test such as the ECG represents an attractive tool, and the ability of AI to extract hidden patterns from ECG signals holds particular promise for earlier detection and triage.

The current body of evidence, however, has several limitations that temper confidence in translation to practice. A key issue is the use of non‐equivalent reference standards across studies. At the more robust end, some studies employed invasive haemodynamics or guideline‐concordant echocardiographic definitions, closely aligned with contemporary clinical practice. In contrast, others relied on simplified preserved‐EF symptom‐based definitions, LVDD surrogates, composite scores or administrative coding, all of which are more vulnerable to misclassification and may inflate performance estimates. Study populations were also variable, ranging from invasive cohorts and case–control series to very large hospital datasets. Small and highly selected samples limit generalizability, while larger registry studies risk diagnostic inaccuracy if coding is relied upon [27]. These differences likely contribute directly to the observed heterogeneity and may lead to overly optimistic estimates of diagnostic performance in controlled or highly selected datasets compared with real‐world populations. Importantly, evidence derived from studies using invasive or guideline‐concordant reference standards should be prioritized when assessing clinical readiness, as it provides a more valid representation of true diagnostic performance than studies based on administrative coding or simplified definitions.

A further limitation is the reliance on AUROC as the primary performance metric. While AUROC reflects discrimination, it does not capture calibration or clinical decision utility, particularly in imbalanced conditions such as HFpEF. Reporting of calibration was limited, precision–recall analyses were rarely performed and clinically actionable thresholds were inconsistently defined or externally validated. As a result, high AUROC values may not translate into clinically meaningful performance, especially where false positives and false negatives have important consequences. Accordingly, the current literature risks conflating statistical discrimination with clinical usefulness, and greater emphasis on calibration, decision‐curve analysis and threshold‐based performance is needed before implementation. Importantly, no included studies evaluated AI‐ECG within prospective clinical pathways or demonstrated improvements in patient‐centred outcomes, limiting conclusions regarding real‐world clinical effectiveness.

From an AI perspective, methodological rigour was often lacking. Several studies used small training sets such as Gao et al. [18] and Kavas et al. [21], applied imbalance correction without recalibration or failed to assess calibration altogether. External validation was inconsistently performed, and when reported, discrimination was often lower than in internal testing, underscoring concerns about reproducibility, as seen in Kwon et al. [23] and Lee et al. [24]. Accordingly, reports of very high or near‐perfect performance in smaller or methodologically limited studies should be interpreted with caution, as such findings are unlikely to be reproducible in real‐world settings and may reflect overfitting rather than true diagnostic capability. This is particularly relevant in the context of the high pooled AUROC, as performance attenuation in external datasets suggests that real‐world effectiveness may be meaningfully lower than summary estimates imply. Importantly, risk‐of‐bias assessments directly informed our interpretation of model performance. Studies judged to be at higher risk of bias, particularly those with small sample sizes, inadequate validation strategies or unclear data separation, were considered less reliable indicators of true clinical performance. In contrast, studies with external validation, larger and more representative populations and guideline‐concordant reference standards were given greater weight when assessing the potential clinical utility of AI‐ECG. Reporting practices also varied. Some studies reported AUROC without confidence intervals, limiting precision estimates and precluding inclusion in quantitative synthesis, for example, Kavas et al. [21] and Lin et al. [25]. Others reported only AUROC curves without providing clinically relevant thresholds such as sensitivity and specificity at a defined cut‐off, making it difficult to translate findings into practice. In addition, very few studies incorporated robust interpretability analyses, and those that did primarily relied on visualization‐based methods such as saliency or attention maps, which highlight regions of the ECG contributing to model predictions but do not necessarily provide mechanistic insights. Finally, we used AUROC pooling rather than a hierarchical bivariate model because most studies did not report threshold‐level sensitivity and specificity. Although this facilitates synthesis across diverse studies, it may overstate usefulness at real‐world thresholds where sensitivity, specificity and PPV are critical, and it constrains evaluation of clinically actionable cut‐offs and calibration. These methodological weaknesses were reflected in the relatively high risk of bias identified by the QUADAS‐AI tool. In addition, although reduced‐lead and smartphone‐based ECG approaches offer potential advantages in scalability, their diagnostic performance appears less consistent than that of standard 12‐lead models and is supported by fewer, smaller and methodologically limited studies, limiting confidence in their current clinical applicability.

Looking forward, future studies should address these gaps by adopting standardized HFpEF definitions aligned with international guidelines, enrolling large and representative populations that include patients with common comorbidities such as atrial fibrillation and performing robust external validation across diverse healthcare systems. Methodological transparency should be strengthened, with consistent reporting of AUROC confidence intervals, calibration and clinically relevant decision thresholds, alongside adherence to established AI reporting frameworks. Greater emphasis on biologically grounded validation is also required, particularly through correlation of AI‐ECG outputs with echocardiographic parameters and invasive haemodynamic measurements, to enhance interpretability and translational relevance. In parallel, more rigorous interpretability approaches may help build clinician trust and provide mechanistic insights into HFpEF. Finally, pragmatic implementation studies are needed to define how AI‐ECG can be integrated into clinical pathways, including whether it could function as a screening or triage tool to guide further diagnostic testing.

## 5. Conclusion

AI‐enhanced ECG represents a promising and scalable approach for the detection of HFpEF, a condition that continues to present major diagnostic challenges. The available evidence suggests good discriminatory performance, and the widespread availability of ECG provides a unique route to earlier recognition and better targeting of downstream investigations. However, the current evidence base is predominantly retrospective and single centre, with substantial methodological heterogeneity, high risk of bias and variation in the clinical validity of reference standards. Importantly, no prospective, outcome‐based studies have demonstrated improved patient outcomes or evaluated AI‐ECG within real‐world clinical pathways. Accordingly, its role in screening or triage remains uncertain and cannot yet be recommended for routine clinical use. With further development and validation, AI‐ECG may have the potential to support earlier identification, enhance risk stratification and help close one of the most persistent diagnostic gaps in contemporary heart failure care.

## Funding

This research received no external funding.

## Conflicts of Interest

The authors declare no conflicts of interest.

## Supporting Information

Additional supporting information can be found online in the Supporting Information section.

## Supporting information


**Supporting Information** Supporting Information 1–systematic search strategy; Supporting Information 2–PRISMA flow chart; Supporting Information 3a–QUADAS‐AI score summary table; Supporting Information 3b–QUADAS‐AI score detailed table.

## Data Availability

The data that support the findings of this study are available from the corresponding author upon reasonable request.
