# Supplementary material for: Artificial Intelligence–Enhanced Electrocardiography for the Diagnosis of Heart Failure With Preserved Ejection Fraction: A Systematic Review and Meta‐Analysis
Source: Cardiol Res Pract. 2026 Jun 4;2026:4994662. doi: 10.1155/crp/4994662 (PMC13238258; doi:10.1155/crp/4994662)
Supplement: Supplementary file 1 — Supporting Information Supporting Information 1–systematic search strategy; Supporting Information 2–PRISMA flow chart; Supporting Information 3a–QUADAS‐AI score summary table; Supporting Information 3b–QUADAS‐AI score detailed table. [file CRP-2026-4994662-s001.docx]

# Supplementary material 1 – Systematic search strategy

## PubMed (MEDLINE)

("Artificial Intelligence"[Title/Abstract] OR "Machine Learning"[Title/Abstract] OR "Deep Learning"[Title/Abstract] OR "Neural Network*"[Title/Abstract])
AND
("Electrocardiography"[MeSH] OR "Electrocardiogram"[Title/Abstract] OR "ECG"[Title/Abstract] OR "EKG"[Title/Abstract])
AND
("Heart Failure with Preserved Ejection Fraction"[Title/Abstract] OR "HFpEF"[Title/Abstract] OR "Diastolic Dysfunction"[Title/Abstract] OR "Left Ventricular Diastolic Dysfunction"[Title/Abstract] OR "Heart Failure, Diastolic"[MeSH])

## Embase

('artificial intelligence':ab,ti OR 'machine learning':ab,ti OR 'deep learning':ab,ti OR 'neural network*':ab,ti)
AND
('electrocardiography'/exp OR 'electrocardiogram':ab,ti OR 'ecg':ab,ti OR 'ekg':ab,ti)
AND
('heart failure with preserved ejection fraction':ab,ti OR 'HFpEF':ab,ti OR 'diastolic dysfunction':ab,ti OR 'left ventricular diastolic dysfunction':ab,ti OR 'heart failure, diastolic'/exp)

## Web of Science

TS=("artificial intelligence" OR "machine learning" OR "deep learning" OR "neural network*")
AND
TS=("electrocardiogram" OR "ECG" OR "EKG" OR "electrocardiography")
AND
TS=("heart failure with preserved ejection fraction" OR "HFpEF" OR "diastolic dysfunction" OR "left ventricular diastolic dysfunction" OR "diastolic heart failure")

## IEEE Xplore

("artificial intelligence" OR "machine learning" OR "deep learning" OR "neural network")
AND
("electrocardiogram" OR "ECG" OR "EKG")
AND
("heart failure with preserved ejection fraction" OR "HFpEF" OR "diastolic dysfunction" OR "left ventricular diastolic dysfunction")

**Supplementary material 2 - PRISMA flow chart**


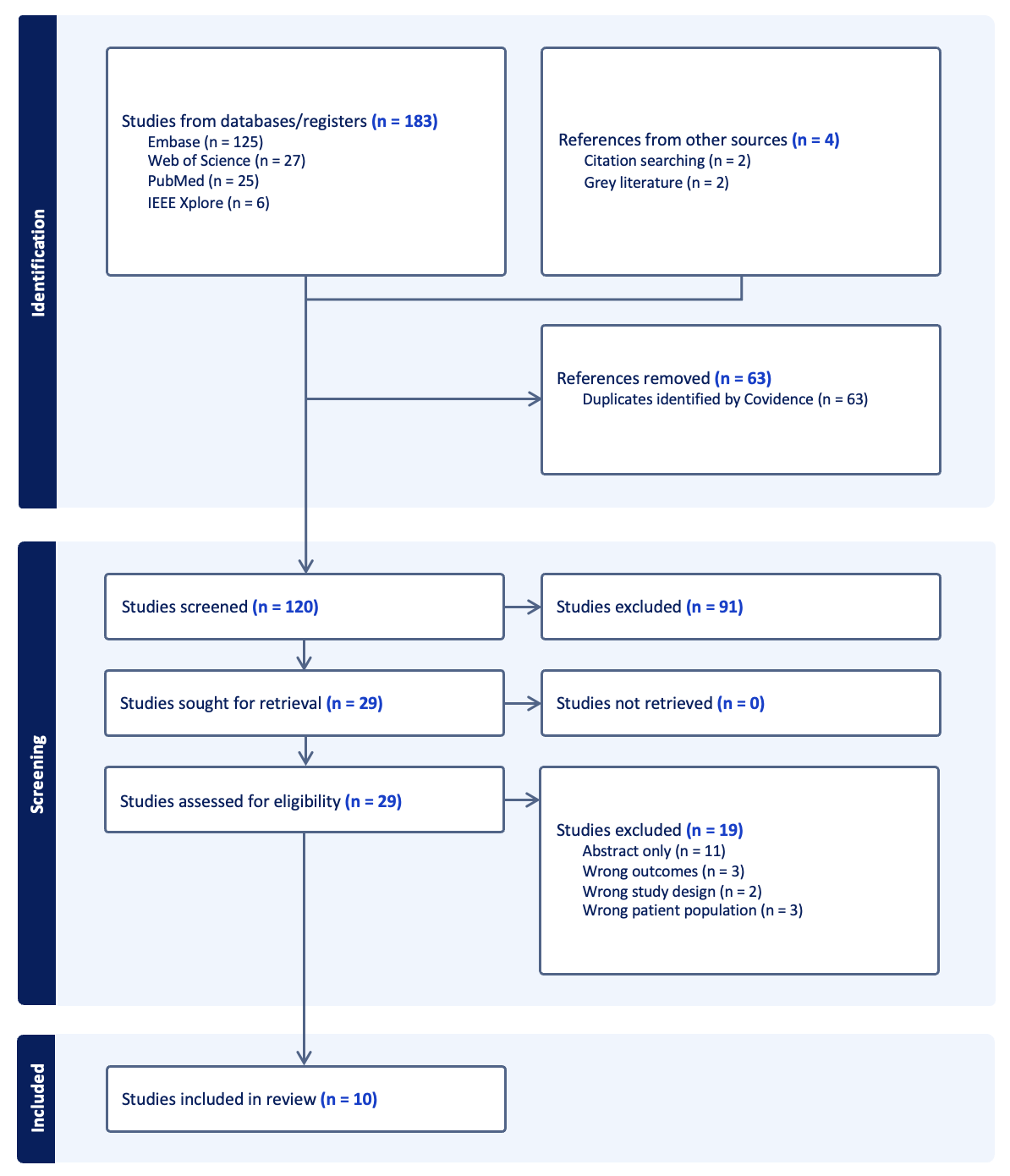


**Supplementary material 3a – QUADAS-AI score – Summary table**

|  | **Risk of Bias** | | | | **Applicability concerns** | | |  |
| --- | --- | --- | --- | --- | --- | --- | --- | --- |
| **Study** | **Patient selection (case-control, consecutive?)** | **Index test conduct & analysis (blinding, leakage, pre-specification)** | **Reference standard** | **Flow & timing (interval; exclusions)** | **Patient selection** | **Index test** | **Reference standard** | **Overall risk** |
| Gao (2025) | Low | Moderate | Low | Low | Moderate | Low | Low | Moderate |
| Hong (2025) | Low | Moderate | Low | Low | Moderate | Low | Low | Moderate |
| Karabayir (2024) | High | Moderate | High | Moderate | High | Low | High | High |
| Kavas (2022) | High | High | High | High | High | High | High | High |
| Kuznetsova (2022) | Low | High | Low | Low | Moderate | Moderate | Low | High |
| Kwon (2021) | Low | Moderate | Low | Low | Low | Low | Low | Moderate |
| Lee (2024) | Low | Moderate | Moderate | Low | Low | Low | Low | Moderate |
| Lin (2021) | High | Moderate | Low | Low | High | Moderate | Low | High |
| Schlesinger (2022) | Moderate | Moderate | Low | Low | Moderate | Low | Low | Moderate |
| Unterhuber (2021) | Moderate | Low | Low | Low | Moderate | Low | Low | Moderate |

Supplementary Material 3A: Simplified QUADAS-AI risk-of-bias summary across included studies. Risk-of-bias domains are presented using a traffic-light format (low, moderate, high). Overall risk of bias was assigned according to the highest-risk domain, in line with QUADAS-AI guidance.

**Supplementary material 3b – QUADAS-AI score - Detailed**

|  | **Risk of Bias** | | | | **Applicability concerns** | | |  |
| --- | --- | --- | --- | --- | --- | --- | --- | --- |
| **Study** | **Patient selection (case-control, consecutive?)** | **Index test conduct & analysis (blinding, leakage, pre-specification)** | **Reference standard** | **Flow & timing (interval; exclusions)** | **Patient selection** | **Index test** | **Reference standard** | **Key reasons (short)** |
| Gao (2025) | **Low**:  Cohort A retrospective, Cohort B consecutive prospective patients | **Moderate**:  No clear blinding; index test applied before knowing catheterisation results; architecture pre-specified but not fully detailed | **Low**:  Invasive LVEDP (>12 mmHg) reference, strong though not universal standard | **Low**:  ECG within 24 h of catheterisation; exclusions | **Moderate**: Single-centre Chinese cohort, narrow age (18–75) | **Low**: Standard 12-lead ECG, broadly applicable | **Low**:  Invasive LVEDP is robust though cut-off >12 mmHg slightly non-standard | Hybrid CNN-LSTM trained on small dataset (238) with ~163k parameters; external validation performed but limited (n=117). No interpretability analysis possible due to model design. Risk of overfitting given sample size. |
| Hong (2025) | **Low:**  Consecutive hospital cohort with clear inclusion/exclusion | **Moderate:**  retrospective split, no blinding reported, no external validation | **Low**:  HFA-PEFF ≥5 standard applied consistently | **Low**:  ECG within 1 year, sensitivity analysis at 3 months | **Moderate**:  single-center, may limit generalisability | **Low**:  standard 12-lead ECGs used | **Low:**  guideline-based scoring system used | Prognostic associations reported; good internal validation, but no external validation, calibration, or interpretability analysis included. |
| Karabayir (2024) | **High:**  Registry-based, non-consecutive, ICD-coded HFpEF | **Moderate:**  Internal + external validation, thresholds not pre-specified | **High**:  HFpEF defined as EF ≥50% + ICD code; not guideline-based | **Moderate:** Unclear ECG–echo interval, some exclusions noted | **High**:  non-representative, prevalence distortion | **Low**:  moderate – modern AI model, but blinding/reporting limited | **High**:  reliance on ICD coding undermines reference standard validity | Very large dataset with both internal and external validation; incorporated interpretability (Grad-CAM++), but outcome definition weak and risk of mislabeling high. |
| Kavas (2022) | **High**:  small diagnostic case–control dataset, multiple segments per subject | **High**:  risk of leakage (record-level split), no blinding, thresholds not pre-specified | **High**:  HFpEF = LVEF ≥50% + symptoms; no diastolic indices/NP | **Unclear/High**: Echo vs ECG timing not specified; minimal detail | **High**:  Small, non-representative spectrum | **High**:  Risk of overfitting, leakage | **High**:  Pragmatic definition, not guideline-based | Traditional ML on engineered features, best k-NN model reported “100% accuracy” on internal split, suggesting overfitting. Small sample and repeated measures undermine robustness. No calibration, no clinical interpretability. |
| Kuznetsova (2022) | **Low:** prospective, consecutive adults; predefined exclusions; two-phase design. | **High:** thresholds/features derived in development phase; limited pre-specification; no AUROC CI in validation. | **Low:**  LVDD by guideline echo (≥3/4 criteria) with preserved EF; blinded readers. | **Low:**  echo then ECG immediately after; small quality exclusions. | **Moderate**: limited to single Russian center, but population not highly selected | **Moderate:** single-lead smartphone device, limited generalisability. | **Low:** guideline echo reference. | Smartphone-case single-lead ECG (CardioQVARK®) with spectral/wavelet processing; algorithm based on 4 derived features (QTc, QRSfi, Tpeak, Toffs). No multicenter external validation, small validation cohort. Very high reported accuracy raises concerns about optimism and reproducibility. |
| Kwon (2021) | **Low:**  consecutive cohort from 2 hospitals, large dataset | **Moderate**:  DLM pre-specified, but no prospective blinding to clinical outcomes; some risk of information leakage not fully addressed | **Low:**  Echo-based LVDD + symptoms / signs with EF ≥50% | **Low:**  ECG and echo within 1 week; minimal exclusions | **Low:**  Broad population, reflects clinical use | **Low:**  Used raw ECG across leads; generalisable | **Low:**  Echo guideline-based definition of HFpEF | Developed ensemble DLM using ECG + demographics, large training set (32k ECGs). Internal and external validation performed, with strong AUC (~0.87). Interpretability explored using saliency maps. Limitations: retrospective, Korea-only, no prospective testing, limited reporting on calibration. Model emphasised QRS and T-wave regions. |
| Lee (2024) | **Low:**  Very large consecutive cohort (>270k), no exclusions | **Moderate**:  ResNet-18 CNN pre-specified, but no blinding; internal hold-out only, no external validation | **Moderate:** diastolic grading by echo, but aggregated to binary outcome; potential misclassification | **Low:**  ECG–echo within 14 days (mean 0.5 d); minimal exclusions | **Low:**  Diverse clinical population | **Low:** Standard 12-lead ECG; highly applicable | **Low:**  Widely accepted echo guideline definition | Trained >200k ECGs, used windowed input method. Reported high AUC (~0.91 for filling pressure). Extensive subgroup analyses including indeterminate-by-echo patients. Mortality associations provided. AI methods strong (ResNet, thresholds by Youden index) but lack of external validation and reliance on single-centre echo grading limit generalisability. |
| Lin et al (2021) | **High**:  Single cohort of 2,206 military males, highly selected, not general population | **Moderate**:  models internally developed and tested with 3:1 split, SMOTE oversampling; risk of data leakage not explicitly excluded; no blinding reported | **Low:**  ASE/EACVI echo diastolic dysfunction criteria | **Low:**  ECG and echo same day; minimal exclusions | **High**:  all young, healthy military men, limits generalisability | **Moderate**: ECG features extracted only, not raw signals; conventional ECG, but heavy pre-processing | **Low:**  guideline-based echo reference | Internal 3-fold cross-validation with SMOTE to address class imbalance, but no external validation performed. Very low LVDD prevalence (4.3%) increases risk of spectrum effects. Hyperparameters tuned on training folds only, but details limited. |
| Schlesinger (2022) | **Moderate**:  single tertiary center cohort of RHC patients, enriched with HF/transplant, not consecutive general population | **Moderate**:  ECG and RHC paired within 24 h but exact blinding not reported; pre-spec thresholds for mPCWP >15 mmHg; risk of data leakage mitigated by patient-level splits | **Low:**  invasive PCWP >15 mmHg as gold standard | **Low:**  ECG and RHC same day; reasonable exclusions | **Moderate:** tertiary RHC cohort not general screening | **Low:**  standard CNN applied to ECG | **Low:**  invasive haemodynamics appropriate | Developed RHCNet, a CNN with pre-training on >240k ECGs and fine-tuning on 5,390 RHC-linked ECGs. Innovative use of an “unreliability score” to flag poor predictions. However, no external multicenter validation, reliance on single-site data, and black-box interpretability remain concerns. Saliency maps showed focus on diastolic phase, but interpretability limited. |
| Unterhuber (2021) | **Moderate:** derivation cohort = patients with dyspnoea and CAD workup, not consecutive; selection bias likely | **Low:**  CNN with train/val/test split; withheld test set; threshold set in derivation; external validation performed | **Low:**  ESC guideline-based HFpEF incl. NT-proBNP and echo indices; robust | **Low:**  ECG, echo, labs collected at index visit; minimal exclusions | **Moderate:**  CAD-suspected cohort and external “at risk” volunteers may not represent general HFpEF | **Low:** standard 12-lead ECG, feasible in practice | **Low:**  guideline-based ESC HFpEF definition with NP included | CNN trained on 77k ECG segments, validated in independent screening cohort; strong external validation, but preprocessing (image filters, segment splitting) limits interpretability. |

Legend: Low = low risk of bias; High = high risk of bias; Moderate = moderate risk of bias; Unclear = insufficient reporting.
